# Early Prediction of Sepsis Onset Using Neural Architecture Search Based on Genetic Algorithms

**DOI:** 10.3390/ijerph19042349

**Published:** 2022-02-18

**Authors:** Jae Kwan Kim, Wonbin Ahn, Sangin Park, Soo-Hong Lee, Laehyun Kim

**Affiliations:** 1 Center for Bionics, Korea Institute of Science and Technology, Seoul 02792, Korea; kimjk@kist.re.kr (J.K.K.); sipark@kist.re.kr (S.P.); 2 School of Mechanical Engineering, Yonsei University, Seoul 03722, Korea; 3 Applied AI Research Lab, LG AI Research, Seoul 07796, Korea; wonbin.ahn@lgresearch.ai; 4 Department of HY-KIST Bio-Convergence, Hanyang University, Seoul 04763, Korea

**Keywords:** genetic algorithm, intensive care unit, neural architecture search, sepsis

## Abstract

Sepsis is a life-threatening condition with a high mortality rate. Early prediction and treatment are the most effective strategies for increasing survival rates. This paper proposes a neural architecture search (NAS) model to predict the onset of sepsis with a low computational cost and high search performance by applying a genetic algorithm (GA). The proposed model shares the weights of all possible connection nodes internally within the neural network. Externally, the search cost is reduced through the weight-sharing effect between the genotypes of the GA. A predictive analysis was performed using the Medical Information Mart for Intensive Care III (MIMIC-III), a medical time-series dataset, with the primary objective of predicting sepsis onset 3 h before occurrence. In addition, experiments were conducted under various prediction times (0–12 h) for comparison. The proposed model exhibited an area under the receiver operating characteristic curve (AUROC) score of 0.94 (95% CI: 0.92–0.96) for 3 h, which is 0.31–0.26 higher than the scores obtained using the Sequential Organ Failure Assessment (SOFA), quick SOFA (qSOFA), and Simplified Acute Physiology Score (SAPS) II scoring systems. Furthermore, the proposed model exhibited a 12% improvement in the AUROC value over a simple model based on the long short-term memory neural network. Additionally, it is not only optimally searchable for sepsis onset prediction, but also outperforms conventional models that use similar predictive purposes and datasets. Notably, it is sufficiently robust to shape changes in the input data and has less structural dependence.

## 1. Introduction

Sepsis is a condition in which inflammatory reactions occur all over the body in response to infection. Severe sepsis can lead to sepsis shock or even death; it results in tissue and organ damage, is a major cause of death worldwide, and has a very high mortality rate [1]. Approximately 270,000 people die of sepsis annually in the United States. There has been a steady global increase in the number of sepsis-related incidents, with approximately 30 million cases worldwide and 6 million deaths [2]; however, no precise treatment has been developed. Early prediction and active treatment through diagnosis continue to be the most effective strategies for reducing mortality [3]. Moreover, if sepsis can be prevented by predicting in advance, it is possible to reduce the consumption of medical resources.

A method capable of objectively diagnosing sepsis is required to accurately predict its onset. Sepsis is a condition that presents a systemic inflammatory response rather than originating from a specific pathogen. Thus, the definition and diagnostic criteria for this condition keep changing. The purpose of these changes has been to determine and treat suspected sepsis more quickly. Sepsis-3, defined in 2016, is the latest definition and is simpler than earlier ones [1]. Although Sepsis-3 has become more practical, it is associated with a lower sensitivity than the systemic inflammatory response syndrome (SIRS) [4]. This study defines sepsis from a predictive modeling perspective as SIRS plus infection [5].

This definition was applied to classify cases with and without sepsis. Previous studies primarily used scoring systems and statistical analysis methods, which are more widely applied. The scoring system is rule-based, making it easily applicable and quickly implementable in hospitals. Typical scoring systems include the Sequential Organ Failure Assessment (SOFA), quick SOFA (qSOFA), and Simplified Acute Physiology Score (SAPS) II [6,7,8]. There have been various prediction studies based on these scoring systems; however, they have focused mainly on mortality prediction [9].

Conditions, such as the region or environment, have not been considered because the rules applied to the system have been conservatively determined. Deep learning is being applied to overcome this drawback. Many methods have been proposed for labeling, learning, and utilizing medical image data to locate and detect the presence of disease [10,11,12]. Applying medical time-series analysis is not as effective as applying medical vision. Medical time-series data contain less information than images, have many noise-related and sensor-based errors, and are complex to analyze. Nevertheless, many attempts have been made to improve the predictability using deep learning methodologies based on medical time-series data [13,14,15,16,17,18,19,20]. In addition, research on diagnosis prediction has been conducted using recurrent neural networks (RNNs) and long short-term memory (LSTM) models [14,15,16], and convolutional neural networks (CNNs) have been used for classifying and analyzing time-series data on the image analysis side [17,18]. These deep learning methodologies outperform conventional approaches [19,20]; however, a relatively large amount of information is required from the input data for accurate results, leading to high computational costs.

The performance of deep learning models is typically reliant on the model architecture determined by human insights and assumptions. To overcome this issue, research is underway on neural architecture search (NAS), a method that attempts to automatically explore optimal architectures [21]. The NAS is a representative methodology in automated machine learning that automates the artificial intelligence analysis process. Neural network architecture, as well as weights, are included in the training process of the model. The NAS has outperformed previous deep learning methods in many fields [22,23]; however, it incurs a high computational cost for the navigation process. Hence, we propose a novel NAS methodology with less computational cost and a high search performance by applying a genetic algorithm (GA). Few studies have applied NAS with a GA to medical time-series analysis, and this study may even be the first to apply NAS to that field. The objective of this study is to improve NAS and demonstrate that it can be applied to sepsis prediction. The approach with a fixed neural network structure is difficult to respond to fast data changes. The existing NAS requires a high cost of exploration, which makes its practical application difficult; however, the proposed model can help predict sepsis quickly with an improved approach to these problems.

The proposed model employs NAS with a GA and is aimed at sepsis onset prediction 0–12 h in advance. A denoising autoencoder is applied in the pre-processing stage to denoise the data and improve learning efficiency. The performance of the proposed model was compared with those of conventional scoring systems and simple machine learning—specifically, SOFA, qSOFA, SAPS II, and LSTM. We also compared the results of existing sepsis onset prediction studies by matching the prediction time.

## 2. Methods

### 2.1. Gold Standard

**Figure 1:**
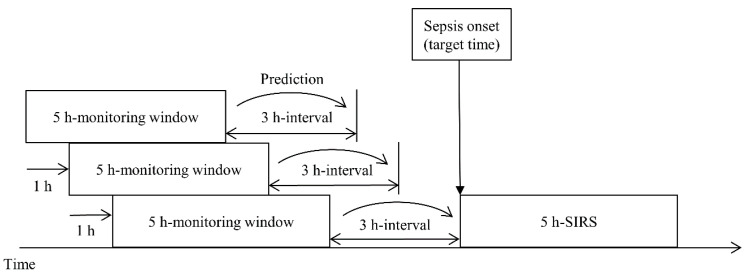
Target time of a prediction model using a sliding window scheme. The prediction time can vary from 0 to 12 h, and 3 h, the primary objective, is for illustrative purposes only.

In this study, we define sepsis onset as the start of a 5 h SIRS interval. Calvert et al. [24] presented the ninth revision of the International Classification of Diseases (ICD-9) diagnostic code and 5 h SIRS as the criteria for sepsis onset; however, there has been a change in the diagnostic codes related to sepsis, and ICD-11 is the most recent revision. Furthermore, because the definition of sepsis keeps changing, it was necessary to redevelop the data used to meet the latest standards. Therefore, we updated the sepsis diagnosis to SIRS and suspected infection and used it as the first criterion. For the second criterion, we defined the starting point of SIRS occurrence as the sepsis onset when SIRS occurred continuously for 5 h and aimed to predict sepsis onset 0–12 h in advance. Figure 1 shows the sliding window process.

### 2.2. Dataset

The Medical Information Mart for Intensive Care III (MIMIC-III) includes medical data in a relational database [25]. It mainly consists of intensive care unit (ICU) data from Beth Israel Deaconess Medical Center in Boston, from July 2001 to October 2012. The data include vital signs, laboratory measurements, care providers, notes, fluid balances, and diagnostic codes. The dataset contains 26 database tables; we used data from the Chartevents, Inputevents, Outputevents, Labevents, and Prescriptions tables for our analysis. Moreover, the Admissions and ICUstays table data were used to confirm the consistency with the data structure. The Chartevents table contains 330 million chart events with approximately 13,000 features, such as the heart rate, temperature, and blood pressure. For example, when a medical team examines body temperature, the time and value are recorded as an event. Table 1 shows information pertaining to the essential features for general consideration, including the number of admissions. There is a difference between the admission and adult patient numbers because one patient can be admitted multiple times. Therefore, there are more hospitalizations than adult patients.

The Chartevents table includes times, features, and results in the form of treatment records. The records were filtered in the pre-processing stage because there were invalid data, and then used for learning. Furthermore, inconsistent units were combined into a unit with the majority of recorded counts. For example, among observations of the same feature, the units of milligrams and kilograms were mixed. In this case, the unit was unified in milligrams. When multiple records existed for the same feature simultaneously with different values, the Labevents table was given priority. If multiple features only have different spacings or are written in similar ways, they were treated as a single feature. Table 2 shows an example of the format displayed in such a case.

The entire dataset contains episodes. An episode is a 5 h period of data corresponding to a monitoring window. Each episode is labeled with sepsis development after the target prediction time when the episode is sampled. Patient data are sampled without being distinguished by the patient in the episode extraction process. Even when extracted from different patients of the same age and gender, the episodes are processed in the same way. Because the episodes are extracted on an hourly basis, there are more episodes than ICU hospitalization records. A patient may have had multiple hospitalizations, and each hospitalization event may have generated multiple ICU records. The missing data rate was measured for all the recorded features, and episodes with fewer than 50% of the features were excluded. The dataset contained 58,976 patients and 5325 sepsis cases before following the above filter conditions. If filtering is first applied to include only adults over the age of 18, the number of patients decreases to 50,799, and the number of sepsis cases becomes 5319. To categorize a sepsis case, a prescription record confirming an infection is necessary; however, even if sepsis occurs, there may be no such infection or SIRS record. After excluding cases with no infection or SIRS record, 32,790 patients and 2724 sepsis cases remained. Each case had a time condition that exceeded 8 h, including monitoring and prediction times.

**Figure 2:**
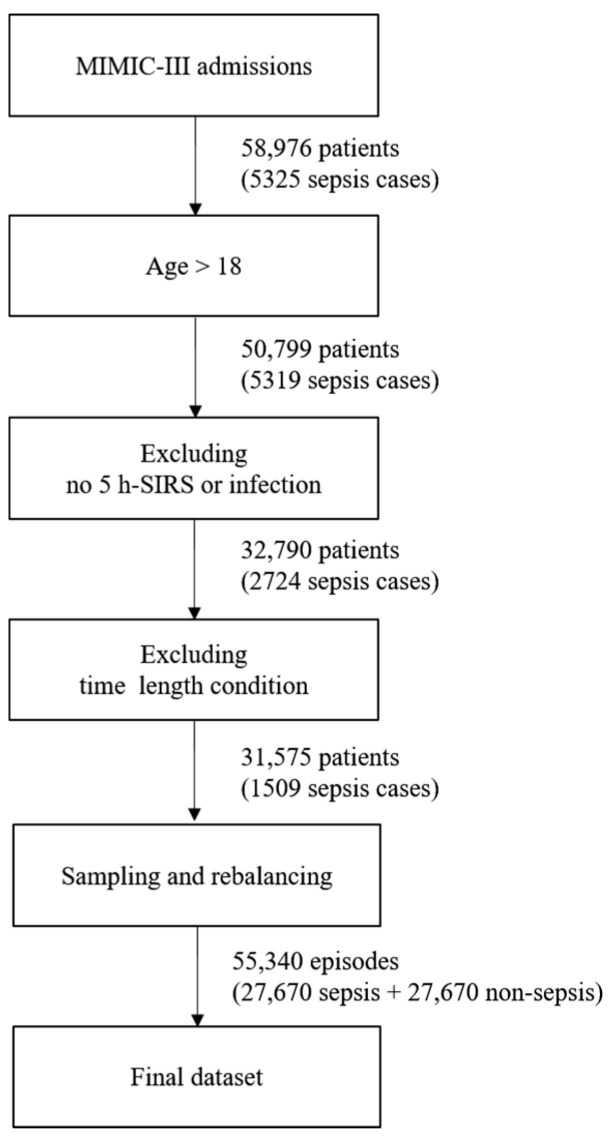
Process of creating the final dataset.

If this condition is applied, up to 31,575 patients and 1509 cases of sepsis remain. Next, a total of 55,340 episodes become datasets by organizing them into 5 h units. If there are more episodes than the number of patients or if the hospitalization time of the patient is 5 h or more, sampling is done while moving forward 1 h. Therefore, multiple episodes may occur; however, there will be a class imbalance problem if this dataset is used as it is. This is because there are only 1500 sepsis episodes out of the approximately 50,000 episodes. To solve the imbalance problem of the class, we applied the random oversampling method to increase sepsis episodes, while decreasing non-sepsis ones by undersampling. The newly created instances are included in the original dataset and constitute the final dataset. Figure 2 shows the process of creating the final dataset, which was split into training, validation, and test datasets for the experiments. The ratio of each dataset was randomly sampled and applied at a 5:3:2 ratio. The validation dataset was intended to prevent overfitting, and the test set was used only for the final performance measurement of the trained model.

### 2.3. Feature Extraction

The features used as input are similar to those used in the InSight [24] model and SOFA, qSOFA, and SAPS II scoring systems. Table 3 shows the features used in each scoring system, some of which are common. In this study, 40 features were selected for the experiments. Although the dataset had 13,000 features, we generally filtered features with a missing rate of less than 80%. The filtered results are almost identical to the features used in the existing work. Hence, we selected the features by referring to the existing studies [2]. The objective was to minimize human intervention; therefore, we did not add derivative features based on conventional medical knowledge.

### 2.4. Imputation and Denoising

An additional pre-processing step was performed to improve the learning efficiency rather than using the cleaned data for learning. The imputation of the missing values helps to convert discrete data into a continuous form and remove noise. Data imputation was performed using a nonparametric Gaussian process regression (GPR), which is a nonparametric kernel-based probabilistic model; it also performs exceptionally well in previous versions of the MIMIC dataset [26].

**Figure 3:**
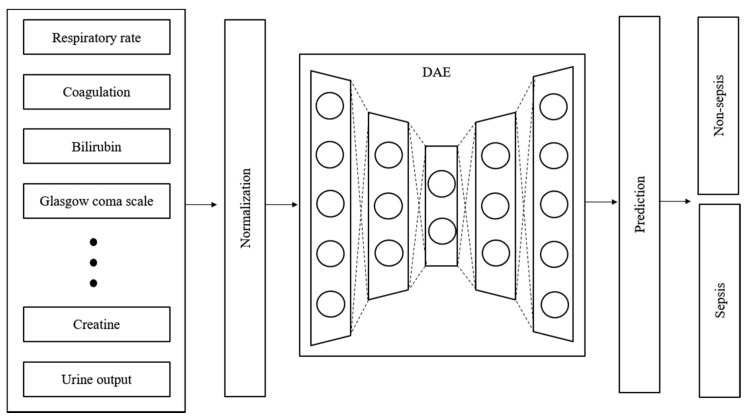
Structure and input data flow of the proposed model.

Time-series data are sensitive to noise, which can increase the prediction error by reducing the learning efficiency of the model. The prediction error can be reduced if a denoising process is applied to the data before learning [27]. Generally, wavelet and viral filter techniques are used as denoising methods; however, the denoising level is determined passively. The autoencoder is a neural network that aims to reproduce itself and was applied to automate this process. Various conditions and restrictions can be applied in this network to change its structure, thus enabling it to create new output and have the desired effect on the original data. Moreover, it can be used for feature recognition because it can be reproduced on the basis of the characteristics of the input data [28]. Figure 3 shows the design of the proposed model. The denoising autoencoder (DAE) was applied to reduce noise in the input data. The raw data used in the experiment were normalized and denoised through the DAE. The trained DAE is an independent module, and all the data inputted to the entire network enter through the DAE.

### 2.5. Proposed Model

**Figure 4:**
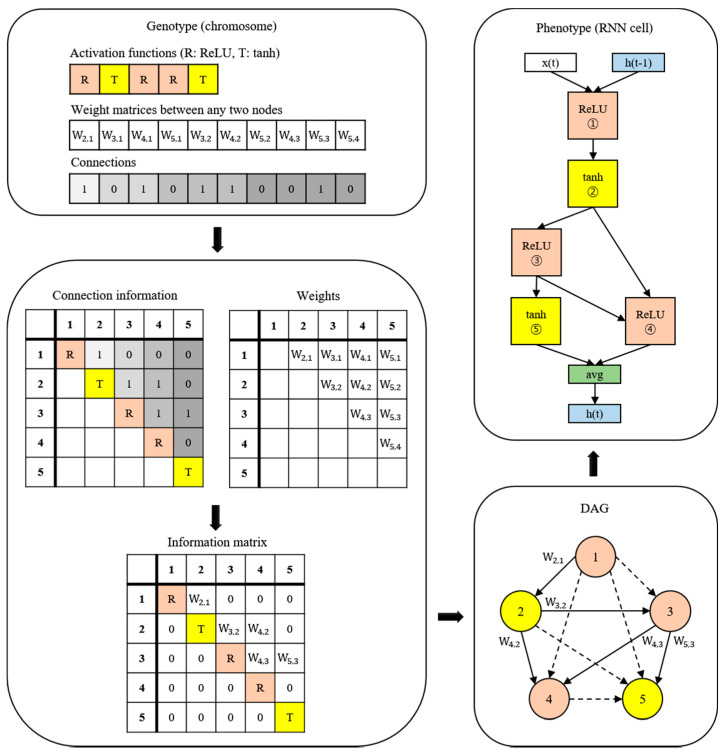
Example of the process of translating from a genotype into a phenotype. The components of a diagonal matrix are the activation functions of each node. The solid arrows in the DAG represent the selected connections.

A new NAS methodology was developed to explore neural architectures for sepsis prediction. The architecture comprises a large RNN network, as shown in Figure 4. The entire model was built by connecting RNN cells, a neural network with N nodes. Therefore, the purpose of the NAS is to search for the optimal RNN cell architecture, not the entire network architecture. An RNN cell comprises nodes which are connected by edges. Each node has one activation function, and the possible activation functions are tanh, ReLU, identity, and sigmoid. If N nodes and four activation functions are allowed, 4^N^ × (N − 1)! cases can be created. Figure 4 shows the architecture and an example of a current cell with five nodes. In this study, the number of nodes in the RNN cell was set to 12. Thus, there are approximately 10^14^ models in the search space. Several strategies can be utilized to reduce the search cost, given that it takes a considerable amount of time to search the entire space. Among these strategies, we used the method of sharing parameters between child networks [22]. The GPU used for this experiment was the NVIDIA TITAN Xp (NVIDIA Corporation, Santa Clara, CA, USA).

The weights are combined with the connection information to generate an information matrix, which is expressed as a directed acyclic graph (DAG). The cell structure is determined based on this DAG. The converted network outputs an operation when a node is inputted and selects a node when an operation is inputted.

**Figure 5:**
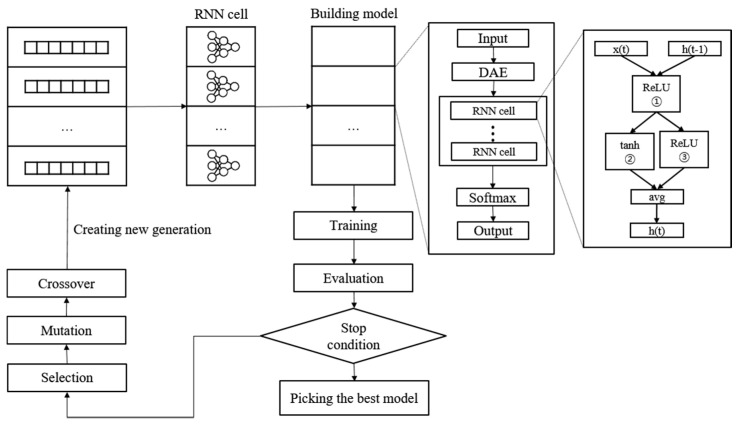
Neural architecture search and training process with genetic algorithm.

Once learned, the shared weights are used for subsequent learning when the same connection occurs in other networks. The GA was used for the controller to create and optimize this RNN cell. The GA has the effect of sharing weights between generations, which can reduce navigation costs [23]. Figure 5 shows the search and training processes of the prediction model. The GA constructs the population, a chromosome group with various genes, and evolves the chromosomes by repeated selection, mutation, and crossover to find the solution [29]. The phenotype and genotype were defined, and conversion was enabled between them to apply this mechanism to the NAS. In other words, the phenotype became an RNN cell, and the genotype became a chromosome. A chromosome contains the activation function information for each node, weight information for all possible edges, and connection information between nodes. Weight information regarding the edge is stored after it is learned; therefore, even if the connection continues to change, it can be learned faster than from scratch. All the child networks can share these stored weights to accelerate the discovery. In the case of the connection information, the connection can only be made to increase the number of nodes to prevent the cycle. A node without output becomes a terminal node, and the average output of the terminal nodes becomes the final output of the cell. The conversion process first converts the genotype into the form of an adjacency matrix, then constructs the DAG that it is based on. The genotype chromosome is converted to an RNN cell because the DAG can be expressed as a neural architecture.

To start a search, we construct the initial population to create the first generation. The population size is set to 100. In other words, a generation comprises 100 chromosomes. Each chromosome transforms into an RNN cell to construct the RNN model. The evaluation of the RNN model is based on the accuracy of the validation set, which is used as the fitness score of the chromosome. Subsequently, we check to determine if the stop condition is met. Training is stopped if the loss of validation in 10 epochs does not decrease or if the total epoch exceeds 200. The learning rate is set to 0.01, and the Adam optimizer is used. Cross-entropy is applied as the loss function. The chromosome with the best performance is selected when it meets the stop conditions. If the stop conditions are not met, a new generation is constructed, and the evolutionary steps are repeated.

The first step in creating a new generation is selection. The parent chromosome is selected as the elite selection using the roulette selection method. Elite selection involves choosing the top 10% of the previous generation of chromosomes as the next generation based on the fitness score. Roulette wheel selection is a method of creating a random number by replacing the fitness score of each chromosome with the area of the roulette wheel, similar to throwing a dart and selecting the chromosome in the area containing the number. Naturally, the higher the fitness score, the larger the area, and the more likely its selection; however, if the highest score is ten times (or more than) the lowest score, the low chromosome is rarely selected. This reduces diversity and makes it easier to reach local optimization. Therefore, when converting to a roulette wheel, the wheel area was adjusted such that the largest area is three times the smallest to select more diverse chromosomes. The selection process was repeated until the remaining 90% were filled, with 10% remaining for the elite chromosomes. The chromosomes selected in this stage form a new generation by creating a child chromosome through the mutation crossover phase.

Crossover is applied to the two chromosomes chosen in the selection step. It is challenging to apply crossover in typical neural networks; nevertheless, genotypes can be easily crossed. The crossover point is randomly selected, and the two chromosomes swap genes with one other after the crossover point. In this study, crossover applies to each property of the genotype. Weights are crossed between weights, and activation functions are crossed between activation functions. The intersection rate was set to 0.7. Mutation applies to a single chromosome. The mutation rate determines whether to apply mutation, and the mutation process randomly selects one gene and changes it to another. The activation function changes to any one of the other remaining functions. For the weight, it changes to a random value. As shown in the above process, the parameters between the chromosomes are shared during the production of a new generation, thus reducing the search cost.

### 2.6. Evaluation

To summarize the experimental process, if data divided into 5 h blocks are inputted, the predictions are made 0–12 h later and subsequently classified on the basis of the predictions. The classification results show whether the predictions are good or bad. The model performance is evaluated with a test set that is not used in the training process. The performance of the proposed model was evaluated using the area under the receiver operating characteristic curve (AUROC) regions of sensitivity, specificity, and receiver operating characteristics. The sensitivity and specificity can be calculated from a combination of true positive (*TP*), true negative (*TN*), false negative (*FN*), and false positive (*FP*).

$$\mathrm{Sensitivity}=\frac{TP}{TP+FN},\;\mathrm{Specificity}=\frac{TN}{TN+FP}$$

The created RNN model was compared with existing scoring systems (SOFA, qSOFA, and SAPS II), machine learning models such as InSight and LSTM, and the results of previous studies [14,20,24,30,31,32,33,34,35,36,37,38,39,40].

## 3. Results

**Figure 6:**
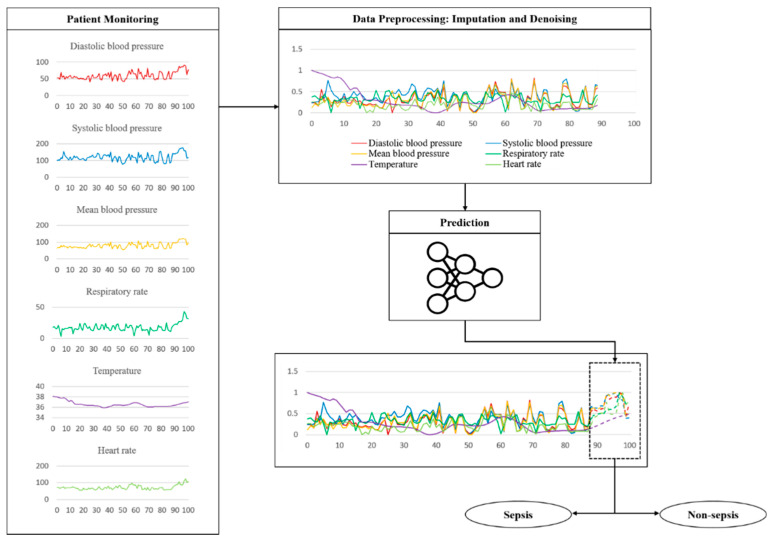
Example of a flow using actually vital data during a test.

The key features related to the vitals used in the experiment are the shape of some actual data and the process of testing the trained model, as shown in Figure 6. The fluctuation is considerable except for the body temperature. These data are sent through a noise removal process using a trained model, followed by a prediction process. Table 4 shows the sepsis onset prediction results which were obtained by the various models, including rule-based scoring systems [14,20,24,30,31,32,33,34,35,36,37,38,39,40]. The existing studies summarized in Table 4 are all sepsis predictions, and the AUROC is included in the evaluation criteria; however, the definition of sepsis and the dataset used in the experiment are different. It is also necessary to consider that the timing and definition of the prediction are slightly different. Only the best results of the existing studies are used in the comparison with the proposed model.

The prediction time was set to 3 h for SOFA, qSOFA, SAPS II, and LSTM. Antibiotic-based treatment requires a certain amount of time and may be successful when sepsis is predicted early. Therefore, the primary purpose of the proposed model was to predict sepsis at least 3 h before the onset. The SAPS II scoring system produced an AUROC of 0.68. The lowest AUROC was 0.63 for SOFA, and qSOFA produced an AUROC of 0.65. The sensitivity (0.65) of SOFA and SAPS II was higher than that (0.61) of qSOFA; however, SOFA produced the lowest specificity of 0.58. The specificity values of qSOFA and SAPS II were 0.75 and 0.77, respectively. qSOFA and SAPS II showed similar results in terms of sensitivity and specificity. LSTM outperformed these scoring systems, with a sensitivity of 0.83; however, the specificity of LSTM was 0.74, lower than that of SAPS II and similar to qSOFA. The AUROC of the LSTM was 0.84, the highest among all the scoring systems. The proposed model showed the highest performance among the compared models, with a sensitivity of 0.93, a specificity of 0.91, and an AUROC of 0.94, based on a 3 h prediction time.

We experimented by varying the prediction time from 0 to 12 h to compare the proposed model with previous studies. Few of the existing studies matched all environments, including datasets and predictive purposes. In particular, this study is the first to predict sepsis using NAS. Nevertheless, we refer to a measure of the performance in the same prediction time. Five of the existing studies, listed in Table 4, used the MIMIC-III dataset. The models that showed better results are InSight [30] and DeepAISE [39]. With a prediction time of 4 h, InSight reported an AUROC of 0.74, and DeepAISE produced an AUROC of 0.87. Our model showed a sensitivity of 0.91, specificity of 0.86, and AUROC of 0.93 for the same prediction time.

**Figure 7:**
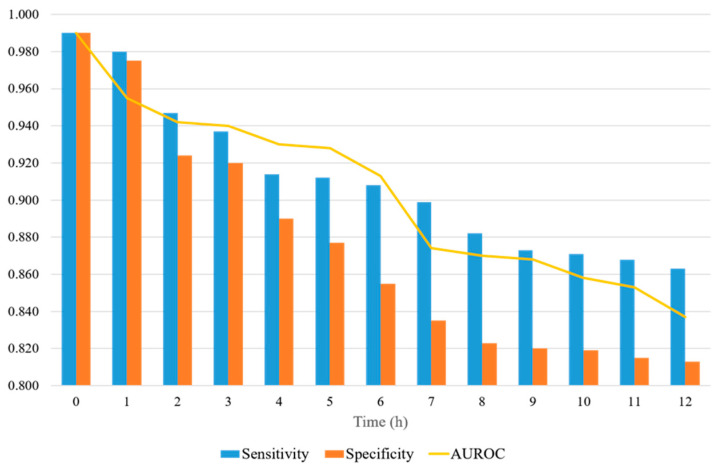
AUROC, sensitivity, and specificity of the proposed model, with the prediction time ranging from 0 to12 h.

Figure 7 shows a representation of the AUROC, sensitivity, and specificity values, with the prediction time ranging from 0 to 12 h. When the prediction time is 2 h, the sensitivity and specificity are significantly reduced. The sensitivity and specificity tend to decrease differently. The sensitivity decreases significantly when the prediction time is between 2 and 8 h, and the decrease is reduced after 8 h. The specificity decreases steadily up to 8 h and then levels off. The specificity decreases more over time than the sensitivity. The sensitivity is always 0.85 or higher, whereas the specificity is 0.80 or higher. When the forecast time is in the range of 3–12 h, the AUROC ranges from 0.94 to 0.83.

**Figure 8:**
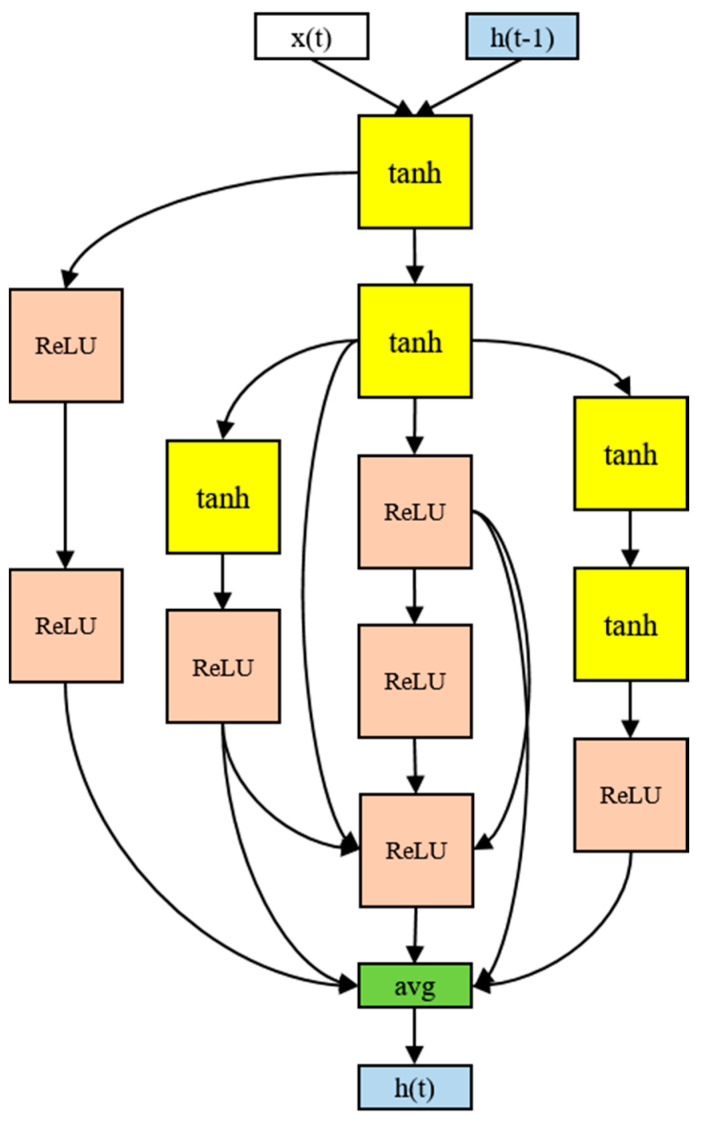
The RNN cell discovered in the proposed model.

The architecture of the RNN cell that produced the best performance in the experiment used 12 nodes, as shown in Figure 8. Although there were four activation functions in the search space, only the tanh and ReLU functions were ultimately selected. The input tended to be tanh and the output ReLU. The final output was the average of the five nodes. In particular, the input part-side connections were distributed, and information was gathered from the multiple nodes in the output part. More irregular skip connections were found than in human-designed models. More skip connections imply more information delivery between the nodes. The 12-node architecture took 16 h to process.

## 4. Discussion

The discovered architecture exhibited a higher predictive performance than conventional methods. A sepsis prediction experiment was conducted on an ICU dataset called MIMIC-III, and the experimental results of the proposed model were compared with those of scoring systems and machine learning methods. The scoring systems produced an AUROC in the range of 0.6–0.7, with most of the results being 0.7 or higher when machine learning methods were applied. The machine learning methods showed better prediction results than the scoring systems. Nevertheless, scoring systems are widely used owing to their ease of application, which is a significant advantage; however, the rules for the scores are conservative, and hence, false alarms occur frequently. Machine learning methods perform well but require a great deal of resources to train the model and may overfit the cohort used for training.

Therefore, it is difficult to compare research conducted using machine learning methodologies. The experimental results depend on the model-training objective, evaluation method, and dataset. AUROC used conditions that were difficult to match, as a rough measurement of the differences in this study. Therefore, the difference in the AUROC should be understood considering the complex experimental environment. Kam et al. [20] used a similar definition and prediction method for sepsis, but with different datasets. In addition, the number of cases used for training was different because of the various pre-processing methods used for the datasets. Existing studies based on the same dataset, namely MIMIC-III, also showed different results for different purposes. Desautels et al. [30] applied the InSight algorithm and obtained an AUROC forecast time of 0.74 for 4 h. Nemati et al. [31] proposed AISE, and Shashikumar et al. [39] proposed DeepAISE, with AUROC forecast times of 0.85 and 0.87 for 4 h, respectively. Moor et al. [33] proposed MGP-TCN, with an AUROC forecast time of 0.86 for 7 h. Studies that used the 2019 Physionet/CinC Challenge dataset, which includes parts of MIMIC-III, were compared. We screened several studies that used this dataset and summarized those that produced high AUROC scores. Li et al. [34] recorded an AUROC of 0.75 for a 12 h prediction, and Yang et al. [36] obtained an AUROC of 0.85 for a 1 h prediction. Li et al. [38] proposed a model based on LightGBM and obtained an AUROC of 0.85 for a 6 h prediction. Rafiei et al. [40] proposed a model called SSP and demonstrated an AUROC of 0.92 for a 4 h prediction. Our model recorded the highest AUROC, based on the same dataset and prediction time, thus demonstrating better performance than conventional scoring systems.

The proposed model automatically generates neural network architectures for prediction, which brings significant advantages in terms of flexibility. In general, the structure of the model relies heavily on data when training deep learning models. Therefore, if the shape and distribution of the data vary, it is difficult to obtain optimal results without changing the model architecture. The proposed model automatically changes its architecture, even if the data are constantly changing, to achieve optimal results. In addition to structural changes, skip connectivity is the main difference between the proposed and human-made models. This means that nodes are not sequentially connected layer-wise but are connected beyond the intermediate node to another node. This technique is used to input information that is diluting backward and reflect it on the nodes close to the output; however, it is challenging to add skip connections manually because there is no standardized skip connection method. Therefore, manually configured models have few skip connections or they are added using a rule. In comparison, the proposed model has many irregular skip connections.

This phenomenon can be explained by assuming that the loss function surface changes smoothly when a skip connection exists [41]. In the absence of skip connections, the solution space of the loss function is irregular, which is likely to cause a local optima problem. Conversely, smooth surfaces are likely to find the best solution with minimal movement. Therefore, this is considered a factor that allows the proposed model to outperform the existing models.

Despite the many advantages of our method, this study has some limitations. First, the computational cost will be high when used in real-world situations if the medical staff updates the model through real-time learning. For real-time learning and updating, the search space needs to be scaled down, or more efficient navigation strategies are required. Second, the GPR used in the data imputation requires a high computational cost, particularly when the number of samples is small and data with high variance and noise are outputted. A process for checking and filtering the outputted values is necessary because some of these output values are not realistic.

## 5. Conclusions

Sepsis is a potentially fatal condition with very high mortality rates. Therefore, early response through prediction is vital. This study proposed and developed a sepsis onset prediction model by applying NAS methodology with a GA optimization process to reduce exploration cost and improve efficiency. The objective was to predict sepsis onset 0–12 h in advance, and this approach was designed to minimize human intervention throughout the process. Therefore, the proposed model was devised to reduce dependence on its structure and be more flexible in response to input data. The results showed that the proposed model outperforms existing methodologies, with an AUROC of 0.94 for a 3 h prediction.

As part of future work, we hope to validate the proposed model and improve its performance on datasets with the most recent definition, Sepsis-3. We also plan to further validate the usefulness of the model using a variety of real-world data. It would be interesting to check whether it would be possible to find optimal structures for sepsis and other medical time-series data.

## Figures and Tables

**Table 1:** Baseline characteristics and essential variables are presented as median values (Q1–Q3).

	Overall
Admission	58,976
Adult patients	38,425
Age	65.86 (52.72–77.97)
Gender (female)	15,409
HR ^1^ (bpm)	84.00 (73.00–97.00)
MAP ^2^ (mmHg)	76.00 (67.33–87.00)
RR ^3^ (cpm)	18.00 (14.00–22.00)
Na (mmol/L)	138.00 (136.00–141.00)
K (mmol/L)	4.10 (3.80–4.60)
HCO_3_ (mmol/L)	24.00 (21.00–26.00)
WBC ^4^ (×10^3^/mm^3^)	11.00 (7.90–14.90)
PaO_2_/FiO_2_ ratio	267.50 (180.00–352.50)
Ht ^5^ (%)	31.00 (26.00–36.00)
Urea (mmol/L)	1577.00 (968.00–2415.00)
Bilirubin (mg/dL)	0.7 (0.40–1.70)

^1^ HR, heart rate; ^2^ MAP, mean arterial pressure; ^3^ RR, respiratory rate; ^4^ WBC, white blood cell count; ^5^ Ht, hematocrit.

**Table 2:** Example of merging when feature id and feature name differ in the same feature.

Feature	ID	Name
Heart rate	211	Heart Rate
	220045	Heart Rate
Temperature	678	Temperature F
	223761	Temperature Fahrenheit
	676	Temperature C
	223762	Temperature Celsius
Systolic blood pressure	51	Arterial BP (Systolic)
	442	Manual BP (Systolic)
	455	NBP (Systolic)
	6701	Arterial BP #2 (Systolic)
	220179	Non-Invasive Blood Pressure systolic
	220050	Arterial Blood Pressure systolic
PaO_2_/FiO_2_ ratio	50821	PO_2_
	50816	Oxygen
	223835	Inspired O_2_ Fraction
	3420	FiO_2_
	3422	FiO_2_ (Meas)
	190	FiO_2_ Set
White blood cells count	51300	WBC Count
	51301	White Blood Cells
Glasgow coma scale	723	Verbal Response
	454	Motor Response
	184	Eye Opening
	223900	GCS—Verbal Response
	223901	GCS—Motor Response
	220739	GCS—Eye Opening

**Table 3:** Features in SOFA, qSOFA, SAPS II, and InSight.

	SOFA	qSOFA	SAPS II	InSight
Age			O	O
Heart rate			O	O
pH				O
Systolic blood pressure		O	O	O
Pulse pressure				O
Temperature			O	O
Respiratory rate				O
Glasgow coma scale	O	O	O	
Mechanical ventilation or CPAP			O	
PaO_2_	O	O	O	
FiO_2_	O	O	O	
Urine output	O		O	
Blood urea nitrogen			O	
Blood oxygen saturation				O
Sodium			O	
Potassium			O	
Bicarbonate			O	
Bilirubin	O		O	
White blood cell count			O	O
Chronic diseases			O	
Type of admission			O	
Platelets	O			
Creatinine	O			
Mean arterial pressure	O			
Dopamine	O			
Epinephrine	O			
Norepinephrine	O			

**Table 4:** Experimental results of the proposed model and summarized existing research results.

Authors	Dataset	Model	Prediction Time	Sensitivity	Specificity	AUROC (95% CI)
Calvert et al. [24], 2016	MIMIC-II	InSight	3 h	0.90	0.81	0.83
Desautels et al. [30], 2016	MIMIC-III	InSight	4 h	0.80	0.54	0.74
Kam et al. [20], 2017	MIMIC-II	LSTM	3 h	0.91	0.94	0.93
Nemati et al. [31], 2018	MIMIC-III	AISE	4 h	0.85	0.67	0.85
Khojandi et al. [32], 2018	Oklahoma State University	RF	0 h	0.99	0.97	0.90
Moor et al. [33], 2019	MIMIC-III	MGP-TCN	7 h	-	-	0.86
Li et al. [34], 2019	2019 PhysioNet/CinC Challenge dataset	CNN+RNN	12 h	-	-	0.75
Scherpf et al. [14], 2019	MIMIC-III	RNN	3 h	0.90	0.47	0.81 (0.79–0.83)
Lauritsen et al. [35], 2020	The Danish National Patient Registry	CNN+LSTM	3 h	-	-	0.86
Yang et al. [36], 2020	2019 PhysioNet/CinC Challenge dataset	XGBOOST	1 h	0.90	0.64	0.85
Bedoya et al. [37], 2020	Duke University Hospital	MGP-RNN	4 h	-	-	0.88 (0.87–0.89)
Li et al. [38], 2020	2019 PhysioNet/CinC Challenge dataset	LightGBM	6 h	0.86	0.63	0.85
Shashikumar et al. [39], 2021	MIMIC-III	DeepAISE	4 h	0.80	0.75	0.87
Rafiei et al. [40], 2021	2019 PhysioNet/CinC Challenge dataset	SSP	4 h	0.85	0.81	0.92
This study	MIMIC-III	SOFA	3 h	0.65	0.58	0.63 (0.59–0.67)
		qSOFA	3 h	0.61	0.75	0.65 (0.62–0.68)
		SAPS II	3 h	0.65	0.77	0.68 (0.66–0.70)
		LSTM	3 h	0.83	0.74	0.84 (0.81–0.87)
		The proposed model	3 h	0.93	0.91	0.94 (0.92–0.96)
			4 h	0.91	0.86	0.93 (0.92–0.94)
			8 h	0.88	0.82	0.87 (0.84–0.90)
			12 h	0.86	0.81	0.83 (0.81–0.85)

## Data Availability

The data used in this study are available on request for access via a process documented at https://mimic.physionet.org/ accessed on 11 January 2022.
